# Structural, Elastic, Electronic and Optical Properties of SrTMO_3_ (TM = Rh, Zr) Compounds: Insights from FP-LAPW Study

**DOI:** 10.3390/ma11102057

**Published:** 2018-10-22

**Authors:** Areej M. Shawahni, Mohammed S. Abu-Jafar, Raed T. Jaradat, Tarik Ouahrani, Rabah Khenata, Ahmad A. Mousa, Khaled F. Ilaiwi

**Affiliations:** 1 Physics Department, An-Najah N. University, P.O. Box 7, Nablus, Palestine; areeg-92@hotmail.com (A.M.S.); raieddd@hotmail.com (R.T.J.); khaled.ilawi@najah.edu (K.F.I.); 2 Ecole Supérieure en Sciences Appliquées, B.P. 165, Tlemcen 13000, Algeria; tarik.ouahrani@gmail.com; 3 Laboratoire de Physique Quantique et de Modélisation Mathématique de la Matière (LPQ3M), Université de Mascara, Mascara 29000, Algeria; khenata_rabah@yahoo.fr; 4 Department of Basic Sciences, Middle East University, Amman 11831, Jordan; amousa@meu.edu.jo; 5 Department of Basic Sciences, Arab Open University, P.O. Box 4375, Ramallah, Palestine

**Keywords:** perovskite, elastic properties, optical properties, FP-LAPW, mBJ

## Abstract

The structural, mechanical, electronic and optical properties of SrTMO_3_ (TM = Rh, Zr) compounds are investigated by using first principle calculations based on density functional theory (DFT). The exchange-correlation potential was treated with the generalized gradient approximation (GGA) for the structural properties. Moreover, the modified Becke-Johnson (mBJ) approximation was also employed for the electronic properties. The calculated lattice constants are in good agreement with the available experimental and theoretical results. The elastic constants and their derived moduli reveal that SrRhO_3_ is ductile and SrZrO_3_ is brittle in nature. The band structure and the density of states calculations with mBJ-GGA predict a metallic nature for SrRhO_3_ and an insulating behavior for SrZrO_3_. The optical properties reveal that both SrRhO_3_ and SrZrO_3_ are suitable as wave reflectance compounds in the whole spectrum for SrRhO_3_ and in the far ultraviolet region (FUV) for SrZrO_3_.

## 1. Introduction

Perovskite structure solids are of great interest in materials science due to their simple crystal structure and their different unique properties such as ferromagnetism, ferroelectricity, superconductivity, thermoelectricity and colossal magneto resistance [1]. Recently, many experimental and theoretical works have been devoted to perovskite oxides RE–TM–O_3_ (RE represents rare earth and TM represents transition metal elements) [2,3,4].

SrTMO_3_ (TM = Rh, Zr) compounds crystallize in the cubic perovskite structure with space group of space *Pm-3m* (# 221). SrTMO_3_ can be described as Sr^2+^ and O^−2^ ions forming a cubic close packed lattice with TM (Rh, Ti) ions occupying the octahedral holes created by the oxygen. The perovskite structure has a three-dimensional net of corner sharing [TMO_6_] octahedral with Sr^2+^ ions in the 12-fold cavities in between the polyhedral [5]. The Sr atom (alkaline earth or rare earth element) is located at (0, 0, 0), the TM atom (transition metal) at (1/2, 1/2, 1/2) and the oxygen atoms sit at face centered positions (1/2, 1/2, 0), (0, 1/2, 1/2) and (1/2, 0, 1/2). A large number of metallic elements are stable in the perovskite structure, if the tolerance factor *t* is in the range of 0.75–1.0 [6].

The elastic, electronic and optical properties of some perovskite compounds were examined by many researchers [7,8,9,10]. In 1992, Roosmalen et al. studied the structure of SrZrO_3_ [9]. In 2009, Baudali et al. [10] computed the structural, optical, electronic and thermal properties of SrTiO_3_ pervoskite cubic structure by using the full potential linearized augmented plane wave (FP-LAPW) method integrated in Wien2k code [11]. In 2011, Daga et al. [12] used the first principle study to calculate the lattice constant of the cubic SrMO_3_ (M = Ti, Zr, Mo, Rh, Ru). They found the lattice constants of SrRhO_3_ and SrZrO_3_ to be 3.932 Å and 4.076 Å, respectively.

In 2016 and 2017, Ali and Rahaman [13,14] used the pseudo potential method integrated in CASTEP code [15] to study the structural, elastic, electronic and optical properties of SrTMO_3_ (TM = Rh, Mo, Ti, Zr, V) compounds of cubic perovskite. They predicted that SrTiO_3_, SrZrO_3_ and SrVO_3_ have a brittle nature, while SrMoO_3_ and SrRhO_3_ have a ductile nature. Moreover, they concluded that SrRhO_3_ has the highest dielectric constant compared to the other compounds. 

SrZrO_3_ compound (with 4*d*-electrons) is of interest because of its high temperature electronic properties applications such as hydrogen gas sensors and fuel cells. SrZrO_3_ with high perfection can be grown and used as laser-host materials. Shende et al. [16] suggested that these materials can be used in high-voltage capacitor applications because of their high breakdown strengths and high dielectric constant. SrRhO_3_ satisfies the conditions for having significant quantum critical fluctuations [17]. A non-Fermi-liquid behavior in transport properties should be observed in SrRhO_3_ in the range where quantum magnetic fluctuations are active [17]. Considering the heaviness of the band structure, these should be observed in transport [17]. Pseudo gap formation, metal-insulator transitions and high-voltage applications are the other significant properties which draw a considerable attention to 4*d* TMO. To the best of our knowledge, no ab initio investigations of the optical properties of these compounds have been reported and no experimental work on the electronic, elastic and optical properties have been studied too.

In the present work, we have studied the structural, electronic, elastic and optical properties of SrTMO_3_ (TM = Rh, Zr) by using FP-LAPW (all electron method) method. The motivation of this work is to improve the calculations and to provide some additional information to the features of SrZrO_3_ and SrRhO_3_ compounds using the all electron method (FP-LAPW).

## 2. Computational Details

The computation has been performed by using FP-LAPW method integrated in Wien2k code [11]. In this method, the unit cell is partitioned into two types of regions: (i) atomic-centered muffin-tin (MT) spheres with radius *R*_α_; and (ii) the remaining interstitial region [18]. The expansion of spherical harmonics is defined within a muffin-tin sphere of radius *R*_MT_ around each nucleus. The Muffin-Tin radius *R*_MT_ values used are 2.5, 1.95, 1.67 atomic units (a.u) for Sr, Rh and O atoms for the SrRhO_3_ compound and 2.5, 1.8 and 1.5 a.u for Sr, Zr and O atoms for the SrZrO_3_ compound. The plane wave cut-off parameter *R*_MT_ × *K*_max_ is equal to 8. The cut-off energy to separate the core states from valence states is set to be −9 Ry. Inside the sphere, Fourier expanded up to *G*_max_ = 14 with a cut-off *l*_max_ = 12 and 35 k-points in the irreducible Brillion zone with a grid of 10 × 10 × 10 Monkhorst-Pack meshes [19] (equivalent to 1000 k points in full Brillion zone) which are used to obtain self-consistency for SrRhO_3_ and SrZrO_3_ compounds to be better than 0.1 mRy. The Perdew-Burke-Ernzerhof–generalized gradient approximation (PBE-GGA) is used for the exchange correlation potential [20], while the modified Becke-Johnson potential (mBJ-GGA) [21] is also used to improve the energy band gap of the herein studied compounds. 

## 3. Results and Discussions

### 3.1. Structural Properties

**Figure 1:**
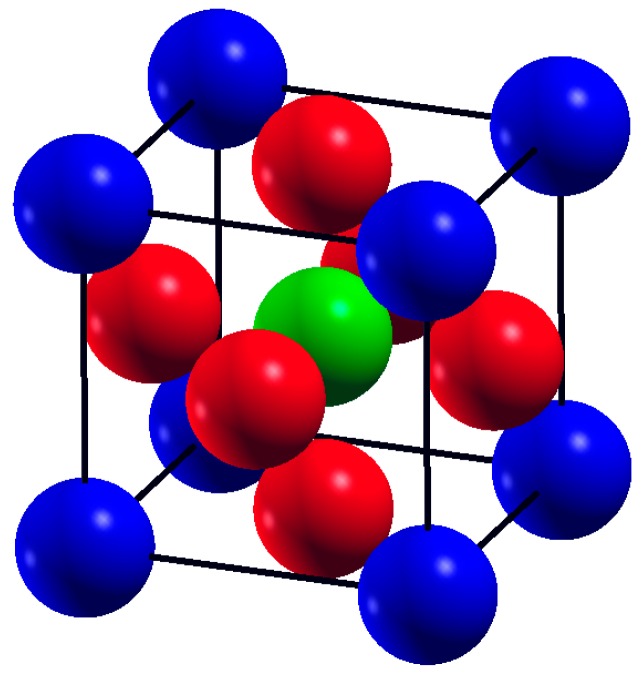
The crystal structure of SrTMO_3_ (TM = Rh, Zr), (blue spheres are Sr atoms, red spheres are Oxygen O atoms and green spheres are TM atoms).

**Figure 2:**
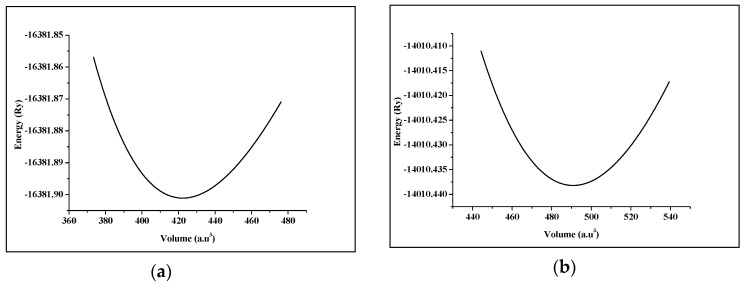
Total energy as a function of the volume of (**a**) SrRhO_3_ compound and (**b**) SrZrO_3_ compound.

Strontium Transition metal oxides (SrTMO_3_) crystallize in the ABO_3_ cubic perovskite structure with the space group *Pm-3m* (221). The crystal structure of SrTMO_3_ (TM = Rh, Zr) is shown in Figure 1. We have optimized the lattice parameters (a) of these compounds by minimizing the total energy. The total energy at different unit cell volumes for SrTMO_3_ (TM = Rh, Zr) is shown in Figure 2, the volume versus energy is fitted to the Murnaghan equation of state [22] to estimate the ground-state properties of these compounds such as the lattice constant (*a*), the bulk modulus (*B*) and the pressure derivatives of the bulk modulus (*B*’). These obtained values are listed in Table 1 together with the available experimental and theoretical data for comparison. In general, the computed structural parameters are in good agreement with experimental and theoretical data available in the literature. More precisely, the lattice parameters of SrRhO_3_ and SrZrO_3_ are found to be 3.976 Å and 4.176 Å, respectively. The present calculations show that the lattice parameter of SrRhO_3_ (SrZrO_3_) is 1.43% (1.63%) larger than the experimental value [23,24]. This guarantees the reliability of the present first-principles computations for the lattice parameters of these compounds using the PBE-GGA method. The calculated bulk modulus for SrZrO_3_ is 2.11% larger than the experimental value [25]. 

### 3.2. Electronic Properties

**Figure 3:**
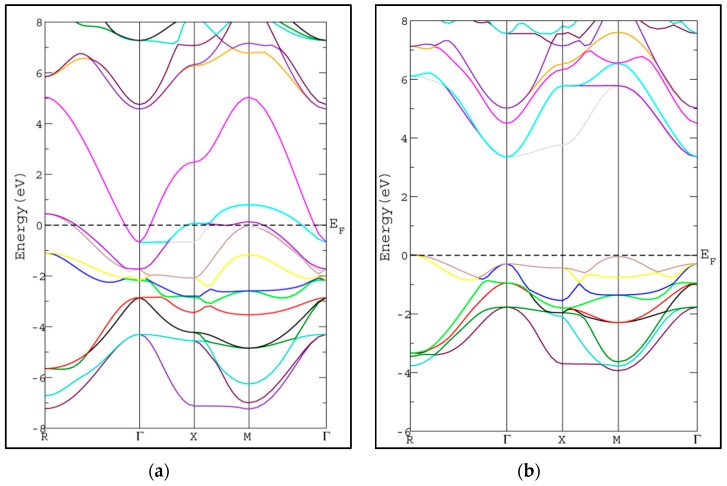
Band structure of cubic pervoskite using PBE-GGA method for (**a**) SrRhO_3_ and (**b**) SrZrO_3_ compounds.

In this section, we study the electronic properties of SrRhO_3_ and SrZrO_3_ via calculating the energy band structure and density of states. The calculated band structures along the high symmetry lines in Brillion-zone of SrRhO_3_ and SrZrO_3_ at zero pressure using PBE-GGA approximation [20] are depicted in Figure 3, where the Fermi level is set at zero eV. In SrZrO_3_, the valence band maximum (VBM) occurs along the M-point symmetry line, while the conduction band minimum occurs along the Γ-point symmetry line with energy band gap 3.69 eV, resulting in an indirect energy band gap (M-Γ) semiconductor.

**Figure 4:**
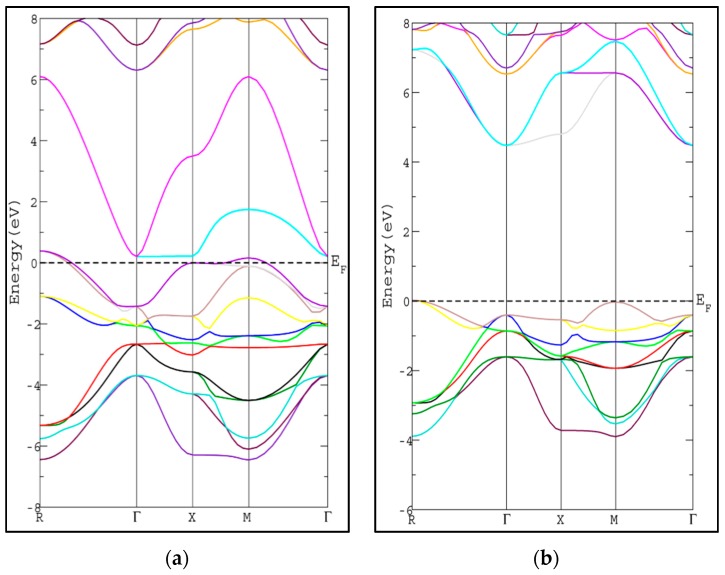
Band structure of cubic pervoskite (**a**) SrRhO_3_ and (**b**) SrZrO_3_ by using (mBJ-GGA) method.

The calculated energy band gap is tabulated in Table 2 along with the available previous theoretical value [26]. The calculated energy gap is larger than the theoretical value by 0.32 eV [26]. To the best of our knowledge, there is no experimental value available to compare with. The usual trend of the modified Becke-Johnson potential (mBJ-GGA) method is enlarging the energy band gap values (*E*_g_) of the semiconductor and insulator materials which makes *E*_g_ comparable to the experimental results [21]. The band structures of SrRhO_3_ and SrZrO_3_ compounds using mBJ-GGA are displayed in Figure 4. For the SrZrO_3_ compound, the minimum energy gap within mBJ-GGA is still indirect with the same direction as the PBE-GGA approach, but its value increases by about 0.85 eV to become 4.54 eV. SrZrO_3_ is classified as an insulator within the mBJ-GGA method. SrRhO_3_ has a metallic nature with no energy gap within the two approaches PBE-GGA and mBJ-GGA. 

**Figure 5:**
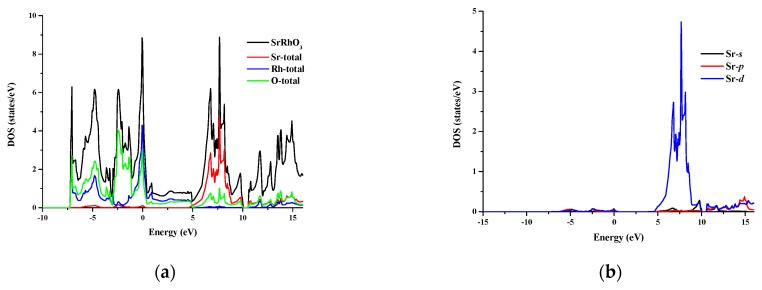

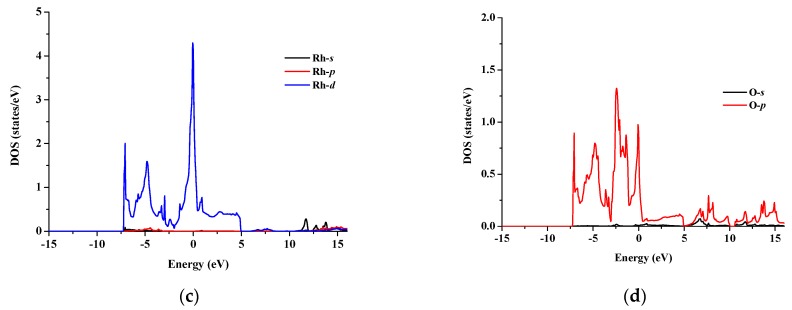
(**a**) Total density of states of SrRhO_3_ compound and partial density of states for (**b**) Sr atom, (**c**) Rh atom and (**d**) O atom in SrRhO_3_ compound.

**Figure 6:**
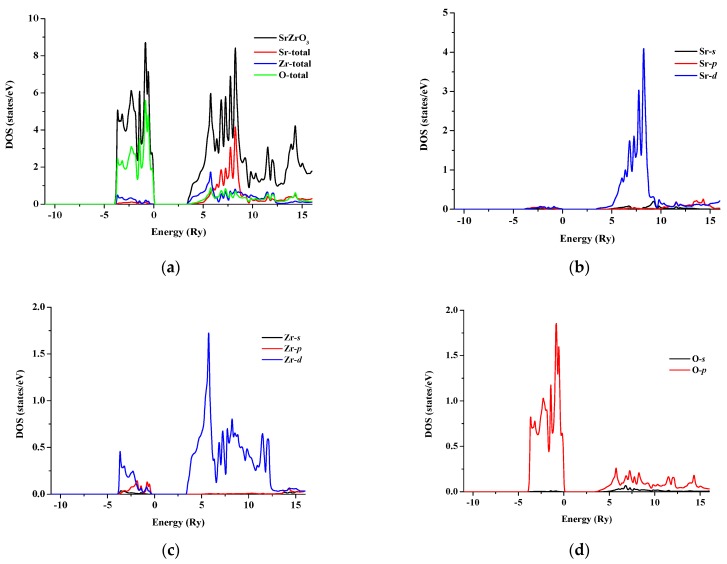
(**a**) Total density of states of SrZrO_3_; Partial density of state for (**b**) Sr atom, (**c**) Zr atom and (**d**) O atom in SrZrO_3_ compound.

The total and partial density of states for SrRhO_3_ and SrZrO_3_ are shown in Figure 5 and Figure 6 for an energy range from −15 to 14 eV and −12.5 to 14 eV, respectively. In SrRhO_3_, the valence band originates from O-*p* and Rh-*d* states. The maximum contribution of the Rh-*d* state is near the Fermi level. In the conduction band, the bands are due to the Sr-*d* with a small contribution from Rh-*d*, O-*p* and O-*s* states. In SrZrO3, the valence band originates from O-*p* with few contribution from Zr-*d*,*p* and Sr-*d* states. In the conduction band, the bands are due to Zr-*d* and Sr-*d* states with a small contribution from O-*p* states.

To make a deeper analysis of the bonding nature of our titled compounds, the wave function obtained from a final Wien2k calculation at the optimal geometry has been analyzed in the critic program (version 1.0). Critic is a full-edged program for the topological analysis of solid-state electron densities.

**Figure 7:**
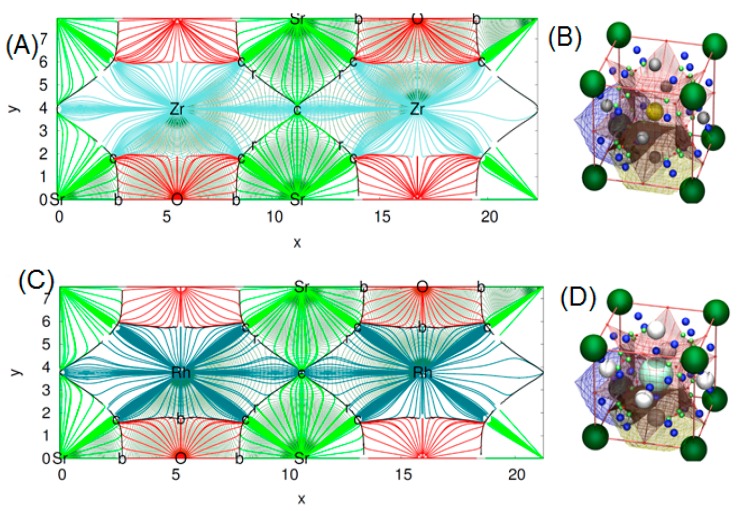
2D map of topological analysis of electron density of (**A**) SrZrO_3_ and (**C**) SrRhO_3_, molecular graph representation of (**B**) SrZrO_3_ and (**D**) SrRhO_3_. In colored trajectories traced out by the electron density gradient, vector field (in electron/bohr^3^) of upper panel: SrZrO_3_ and lower panel: SrRhO_3_ are at their denser plane. Here, the red, dark blue, light blue and green lines refer respectively to O, Rh, Zr and Sr atomic basins. The set of trajectories that terminates at each bond’s critical point (b) defines an interatomic surface. The set of trajectories that originate at the ring critical (r) point defines the perimeter of the interatomic surface. The gradient paths associated with the negative eigenvalues at the (n) point terminate at this CP and define the zero-flux surfaces that partition the crystal into unique fragments (the atomic basins). On the right of the figures, molecular graph representation: the small red balls placed at the Wyckoff’s 3c are the r CPs. The light green balls the b CPs and the dark blue the c cage CPs.

This program uses the quantum theory of atom in molecule (QTAIM) to make a topological analysis of the electronic density (ρ) of a crystal. By means of QTAIM, we can automatically locate all critical points (CP’s) of the electronic density rising from a nil flux of the electron density gradient condition [27]. The procedure implemented in critic is to divide ρ into disjoint regions Ω (basins) through the real space approaches. Here, we can generate topological schemes in two or tridimensional plots. It is clearly shown from the topological distribution of the charge density in Figure 7A, that the charge distribution in the SrZrO_3_ compound is spherically symmetrical. This suggests that all electronic charges are localized around anions—a typical ionic bonding. The same trend is also displayed for the second compound with some small difference in the distribution of ρ due to the charge transfer, which differs in the two titled compounds (see Figure 7C). We also present in Figure 7B,D, the molecular graph of our two perovskites.

**Figure 8:**
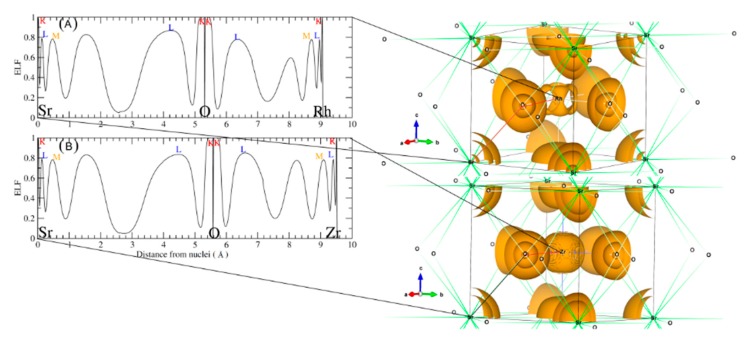
Left panel shows a one dimensional electron localization profile and the right panel shows the 3D isosurface of ELF basins of (**A**) SrRhO_3_ and (**B**) SrZrO_3_ compounds.

These plots were done following the character of Wyckoff’s, bond CP and ring CP family [28]. The form of the atomic basin generated by the CCP point suggests that both SrRhO_3_ and SrZrO_3_ are belonging to the R11 family (see Figure 7B,D) [28]. Here, we can define a topological charge of each atomic basin. As results, *Q*(Sr) = 1.59 electron, *Q*(Rh) = 1.47 electron and *Q*(O) = −1.05 electron for SrRhO_3_, and *Q*(Sr) = 1.61 electron, *Q*(Zr) = 2.49 electron and *Q*(O) = −1.37 electron for the SrZrO_3_ compound. When we compare these charges with the oxidation number corresponding to each of the atoms, we find that the charge transfer is mainly due to the strontium one, with an equal percentage of 80% in both perovskites. However, we note that replacing the cation Rh with electro-negativity of 2.2 by the Zr one with low electro-negativity of 1.4 has a negligible effect on the nature of the bonding. The SrZrO_3_ has a more ionic trend. However, even the constituent cations show different charge transfer (Zr and Rh are transferred by 62% and 37%) and each of them varies as strongly as the oxide ions. The latter adapt their charges according to electro-neutrality requirements. There is a second topological index that has been proposed as a global measurement of the degree of metallicity, the electron density flatness defined as $f=\frac{\rho_{c}^{\min}}{\rho_{b}^{\max}}$ where $\rho_{c}^{\min}$ is the minimal electron density found on the unit cell (it necessarily corresponds to a critical point) and $\rho_{b}^{\max}$ is the maximal electron density among bond critical points. This flatness has values close to one on common metallic compounds and close to zero on localized bonding compounds [29]. The delocalization of electrons (flatness) in the SrRhO_3_ and SrZrO_3_ compounds are respectively equal to 80.61% and 5.62%. This suggests that the global chemical behavior of SrRhO_3_ is metallic, whereas SrZrO_3_ is nonmetallic. To go further in the characterization of the bonds nature of the titled compounds we have employed another tool named the electron localization function (ELF) [30], which is based on a measure of the likelihood of finding an electron in the neighborhood space of a reference electron located at a given point and with the same spin. Taking into account that the homogeneous electron gas has an ELF value of 0.5, valence electrons of metals should deviate very slightly from this quantity. The ELF isosurfaces, as well as the one-dimensional projections of Sr–O–Rh and Sr–O–Zr bond paths of the investigated perovskites are depicted in Figure 8. It is strikingly clear from the plot that O–Zr and O–Rh are different. The integration of population on the ELF attractor of the SrZrO_3_ compound shows two types of Zr–O bonds, the first one has ELF maxima equal to 0.78 and the second to 0.83; the former has the negligible population, but the latter has 0.95 electrons. The integration gives a number of the delocalized attractor as lone pair bonds around the Sr and Zr cations; the population in this attractor varies from 1.4 to 1.1 electrons. Regarding the second compound (SrRhO_3_), no O–Rh bonds have been found, only delocalized lone pair ones with ELF maxima near to 0.5 are reported. Here, we should emphasize that the delocalization of these attractors in the SrRhO_3_ compound provides a reliable measure of the delocalization of wave function and its localization in the SrZrO_3_ compound.

### 3.3. Elastic Properties

In this subsection, we turn our attention to study the mechanical properties of SrRhO_3_ and SrZrO_3_ via calculating their elastic constants. These constants define the properties of material that undergo stress, deform and then recover, returning to its original shape after stress ceases. They have a significant role in finding information about the brittleness, ductility, stiffness and the mechanical stability of the material [31]. The elastic constants require knowledge of the derivative of the energy as a function of the lattice strain. In the case of the cubic system, this strain is chosen in such a way that the volume of the unit cell is preserved. Thus, for the calculation of elastic constants *C*_11_, *C*_12_ and *C_44_*, for these compounds we have used the method developed by Morteza Jamal [32] and integrated in Wien2k code as the IRelast package (Cubic-elastic_13.2). The calculated *C*_ij_ constants are listed in Table 3. Our computed *C*_ij_ data are in reasonable agreement with previous theoretical results. In view of Table 3, it can be noticed that the calculated values of the elastic modulus *C*_11_, which are related to the unidirectional compression along the principal crystallographic directions, are much higher than that of *C*_44_, which represent the resistance to the shear deformation, indicating the weak resistance to the shear deformation compared to the resistance to the unidirectional compression. The mechanical stability of a cubic material requires that its independent elastic constants should satisfy the following Born’s stability criteria [33,34]:

*C*_11_ > 0; *C*_44_ > 0; *C*_11_ + 2*C*_12_ > 0; *C*_11_ > *B* > *C*_12_ (1)

From Table 3, we can see that all required conditions given in the above Equation (1) are simultaneously satisfied, which clearly indicates that the SrRhO_3_ and SrZrO_3_ are mechanically stable.

The three elastic constants *C*_11_, *C*_12_ and *C*_44_ are estimated from first-principles calculations for SrRhO_3_ and SrZrO_3_ single-crystals. However, the prepared materials are in general polycrystalline, and therefore it is important to evaluate the corresponding moduli for the polycrystalline species using the Hill’s approach [35]. In this approach, the effective modulus for polycrystals could be approximated by the arithmetic mean of the two well-known bounds for monocrystals according to Voigt [36] and Reuss [37]. Then, for the cubic system, the shear modulus *S* in the mentioned approximations: Voigt (*V*), Reuss (*R*) and Hill (*H*) are calculated from the elastic constants of the single crystal, in the following form:

(2) $$S_{V}=\frac{1}{5}\left(C_{11}-C_{12}+3C_{44}\right)\;S_{R}=\frac{5C_{44}\left(C_{11}-C_{12}\right)}{4C_{44}+3\left(C-C_{12}\right)}\;S_{H}=\frac{1}{2}\left(S_{v}+S_{R}\right)$$

To compute the Young’s modulus (*Y*), Poisson’s ratio (*ν*), and the anisotropic factor (*A*), the following equations are used, respectively:

(3) $$Y=\frac{9S_{H}B}{\left(S_{H}+3B\right)}$$

(4) $$v=\frac{3B-2S_{H}}{2\left(3B+S_{H}\right)}$$

(5) $$A=\frac{2C_{44}}{C_{11}-C_{12}}$$

The computed bulk modulus, shear modulus, Young’s modulus, Poisson’s ratio and anisotropic factor are listed in Table 3 along with the theoretical results [14,38]. The bulk modulus and shear modulus can be used to measure the material hardness [39]. In general, when $S_{H}$ values increase, the materials become stiffer. From the obtained values of shear modulus $S_{H}$ one can remark that SrZrO_3_ is stiffer than SrRhO_3_.

To categorize the brittle and ductility behaviors of a material, the ratio of the bulk modulus to the shear modulus, *B*/*S*, an empirical relationship related to the plastic and elastic properties of the material, is used. According to Pugh [40], a high *B*/*S* value is associated with ductility, while a low value is consistent with brittleness. The critical value that separates the two behaviors has been determined to be 1.75. The results listed in Table 3 clearly indicate that the SrRhO_3_ (SrZrO_3_) compound has *B*/*S*_H_ ratio higher (smaller) than the critical value of 1.75, which classifies SrRhO_3_ (SrZrO_3_) compound as ductile (brittle) material. In addition, to identify the materials as ductile or brittle, we also applied the Cauchy’s pressure rule defined as the difference between the elastic constants *C*_12_–*C*_44_ [41]. According to this rule, if the Cauchy’s pressure is positive (negative), the material will be ductile (brittle) in nature. As shown in Table 3, It is seen that the Cauchy’s pressure is positive for SrRhO_3_ and negative for SrZrO_3_, confirming the ductile nature for SrRhO_3_ and the brittle behavior for the SrZrO_3_ compound. We may also refer to Frantsevich et al. [42] who distinguishes the ductility and brittleness of materials in terms of Poisson’s ratio (*ν*). According to this rule, if the Poisson’s ratio is less than 0.26, the material will be brittle in nature; otherwise, the material will be ductile. As shown, the computed Poisson’s ratiosare 0.30 for SrRhO_3_ and 0.23 for SrZrO_3_, categorizing SrZrO_3_ as brittle compounds and SrRhO_3_ as ductile compounds. These results exactly agree with the results of the *B*/*S* ratio and Cauchy’s pressure.

The anisotropy factor is an important parameter to measure the degree of materials anisotropy; also, it has a significant usage in engineering science to inspect the potential of micro-cracks in the material [43,44]. For completely isotropic materials, the anisotropy factor *A* takes the value of the unity and the deviation from unity measures the degree of elastic anisotropy. The calculated values of the anisotropic factor *A* are found to be equal to 1.85 for SrRhO_3_ and 0.71 for SrZrO_3_, suggesting that both compounds are anisotropic in nature and SrRhO_3_ is characterized by a profound anisotropy.

### 3.4. Optical Properties

Since the investigated compounds have cubic symmetry, we need to calculate only one dielectric tensor component to completely characterize their linear optical properties. The frequency-dependent complex dielectric function ε(ω) = ε_1_(ω) + *i*ε_2_(ω); where ε_1_(ω) and ε_2_(ω) are the real and imaginary components of the dielectric function, respectively; is known to describe the optical response of the medium at all photon energies E=ℏω, using the formalism of Ehrenreich and Cohen [45].

Complex dielectric function can be derived from the definition given by Hedin [46]

(6) $$\epsilon \left(r,t,r^{\prime },t^{\prime }\right)=\delta \left(r-r^{\prime }\right)\delta \left(t\;-\;t^{\prime }\right)\;-\int P\left(r,t,r^{\prime \prime },t^{\prime }\right)v\left(r^{\prime \prime }\;-\;r^{\prime }\right)dr^{\prime \prime }$$

where *P* is the polarization propagator and is the Coulomb interaction; *P* can be given by the following form:

(7) $$P\left(\omega ,q\right)=\frac{1}{V}\underset{n^{\prime },n,k}{\sum }\frac{f_{0}\left(\varepsilon_{n,k+q}\right)\;-\;f_{0}\left(\varepsilon_{n^{\prime },k}\right)}{\varepsilon_{n,k+q}\;-\;\varepsilon_{n^{\prime },k}\;-\;W}{\left|M_{n,n^{\prime }}\left(k,q\right)\right|}^{2}$$

where *V* is the unit cell volume, *f*_0_ is the Fermi distribution function and ε_k_ is the single particle energy. The matrix element can be given by: *M_n,n’_*(*k*,*q*) = ⟨*u_nk_|*e^−*iq,r*^*|u_n’k_*⟩, *q* is the wave vector of light and it is much smaller than the wave vector of electrons in the system; the matrix elements *M_n,n’_*(*k*,*q*) with small *q* can be given by:

(8) $$M_{m,n}\left(k,0\right)=\delta_{l,n}\;-\;\left(\delta_{l,n}\;-\;1\right)\frac{h\;P_{m,n,k}}{2\pi \left(\varepsilon_{n,k}\;-\;\varepsilon_{m,k}\right)}\;q$$

The sum over *n*’ and *n* must be split into two terms, one with *n*’ = *n* corresponding to intra-band electronic transitions, and the second with *n*’ ≠ *n*, corresponding to inter-band transitions, the intra-band part of the dielectric function ϵ(ω,0) can be given by:

(9) $$\epsilon^{\mathrm{intra}}\left(\omega ,0\right)=1\;-\;lim_{q\to 0}\frac{4{\pi e}^{2}}{V{\left|q\right|}^{2}}\underset{n,k}{\sum }\frac{f_{0}\left(\varepsilon_{n,k+q}\right)\;-\;f_{0}\left(\varepsilon_{n,k}\right)}{\varepsilon_{n,k+q\;}-\;\varepsilon_{n,k\;}-\;\omega }{\left|M_{n,n}\left(k,q\right)\right|}^{2}$$

while the inter band part can be written as

(10) $$\epsilon^{\mathrm{inter}}\left(\omega ,0\right)=-lim_{q\to 0}\frac{4{\pi e}^{2}}{V{\left|q\right|}^{2}}\underset{n,n^{\prime },k}{\sum }\frac{f_{0}\left(\varepsilon_{n^{\prime },k+q}\right)\;-\;f_{0}\left(\varepsilon_{n,k}\right)}{\varepsilon_{n^{\prime },k+q}\;-\;\varepsilon_{n,k\;}-\;\omega }{\left|M_{n,n^{\prime }}\left(k,q\right)\right|}^{2}$$

where *n*’ ≠ *n* in Equation (10).

The imaginary part of the ε(ω) in the long wavelength limit has been obtained directly from the electronic structure calculation, using the joint density of states (JDOS) and the transition moments elements *M_n,n’_*(*k*,*q*):

(11) $$\varepsilon_{2}\left(\omega \right)=\frac{e^{2}\hbar }{\pi m^{2}\omega^{2}}\sum_{v,c}\int_{BZ}{\left|M_{n,n^{\prime }}\left(k,q\right)\right|}^{2}\delta \left[\omega_{n,n^{\prime }}\left(k\right)-\omega \right]d^{3}k$$

The integral is over the first Brillouin zone. The real part of ε(ω) can be derived from the imaginary part using the Kramers–Kronig relations.

(12) $$\varepsilon_{1}\left(\omega \right)=1+\frac{2}{\pi }P\int_{0}^{\infty }\frac{\omega^{\prime }\varepsilon_{2}\left(\omega^{\prime }\right)}{\omega \prime^{2}-\omega^{2}}d\omega^{\prime }$$

where *P* implies the principal value of the integral. The knowledge of the real and imaginary parts of the dielectric function allows the calculation of other important optical functions such as the refractive index *n*(ω), reflectivity *R*(ω), extinction coefficient *k*(ω), energy loss function *L*(ω) and absorption coefficient α(ω) by using the following expressions [47,48,49]:

(13) n(ω)=(12[ε12(ω)+ε22(ω)+ε1(ω)])12

(14) $$R\left(\omega \right)={\left|\frac{\sqrt{\varepsilon \left(\omega \right)}-1}{\sqrt{\varepsilon \left(\omega \right)}+1}\right|}^{2}={\left|\frac{\sqrt{\varepsilon_{1}+i\varepsilon_{2}}-1}{\sqrt{\varepsilon_{1}+i\varepsilon_{2}}+1}\right|}^{2}$$

(15) k(ω)=(12[ε12(ω)+ε22(ω)−ε1(ω)])12

(16) $$L\left(\omega \right)=\frac{\varepsilon_{2}\left(\omega \right)}{\varepsilon_{1}{\left(\omega \right)}^{2}+\varepsilon_{2}{\left(\omega \right)}^{2}}$$

(17) α(ω)=2ωc(ε12(ω)+ε22(ω)−ε1(ω))12

**Figure 9:**
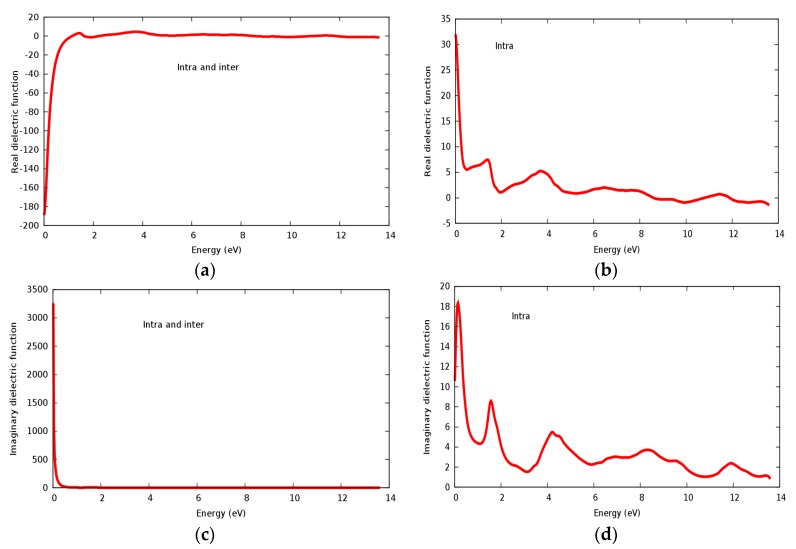
The dielectric function of SrRhO_3_ compound: (**a**,**b**) Real part; (**c**,**d**) Imaginary part.

Real and imaginary parts of the dielectric constant are displayed in Figure 9a–d for the SrRhO_3_ compound. The value of the static real part of the dielectric function ε_1_(0) for the SrRhO_3_ compound within intra and inter band transition in Figure 9a is negative, while the imaginary part of the static dielectric function ε_2_(0) within intra and inter band transition Figure 9c is positive; this implies two important facts: Firstly, the SrRhO_3_ compound has considerable metallic behavior, which agrees with the energy band structure calculations. Secondly, the negative value of (ε_1_) especially in the energy range 0–1.15 eV in Figure 9a and the highly positive value of ε_2_(ω) at the early beginning of Figure 9c, reveal the loss of light transit. Real dielectric constant within intra band transition for SrRhO_3_ in Figure 9b has small peaks; the first two of them are centered at 1.42 eV and 3.73 eV. We see that (ε_1_) for the SrRhO_3_ compound in Figure 9a, it has roots in the 1.15 eV, 1.55 eV, 5.0 eV, 8.45 eV and 13.5 eV. When these roots occur, (ε_1_) = 0, the compound does not respond to incident light, this fact is mainly due to the Plasmon oscillation. The sharp increase in ε_1_(ω) with intra and inter band transition, in Figure 9a, at an energy range 0–1.15 eV, indicates that the compound does not interact with the incident photons at this energy range. The static dielectric constant ε_1_(0) of SrRhO_3_ with inter + intra in Figure 9a and intra band transition Figure 9b are −180 and 32, respectively. Negative value ε_1_(0) of with inter + intra band transition ensures the metallic behavior of SrRhO_3_ compound. By taking only the intra band transition into account as seen in Figure 9b,d, both the real and imaginary parts of dielectric constant have positive values, which indicates a mix of metallic and semiconducting behavior for the SrRhO_3_ compound. This implies that for metallic compounds inter and intra band transitions must be taken into account.

**Figure 10:**
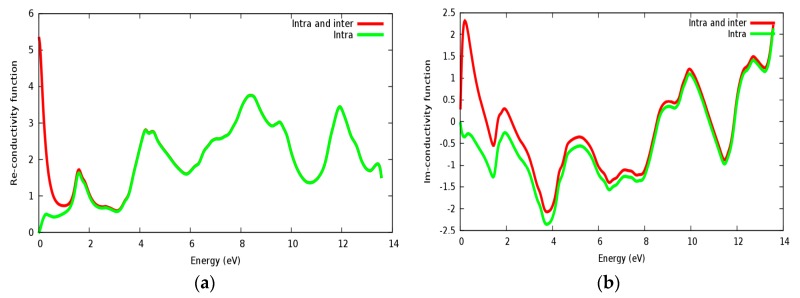
The conductivity function of SrRhO_3_ compound: (**a**) Real part; (**b**) Imaginary part.

Optical conductivity is a quantity depending on the inter band and intra band transitions. In Figure 10a,b, the real and imaginary parts of conductivity are illustrated for the SrRhO_3_ compound, by taking the inter and intra band transition into account; the static real conductivity is high, while it is zero when only intra band transition is taken into account. As the incident light energy increases, the real and imaginary parts of conductivity with the two approaches; intra + inter and intra band transition; both almost have the same behavior.

**Figure 11:**
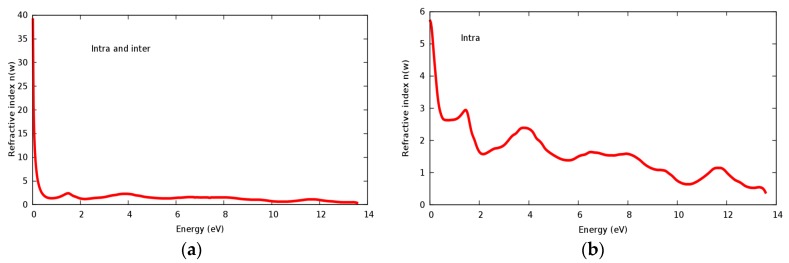
(**a**) Refractive index *n*(ω) within inter and intra; (**b**) Refractive index *n*(ω) within intra for SrRhO_3_ compound.

**Figure 12:**
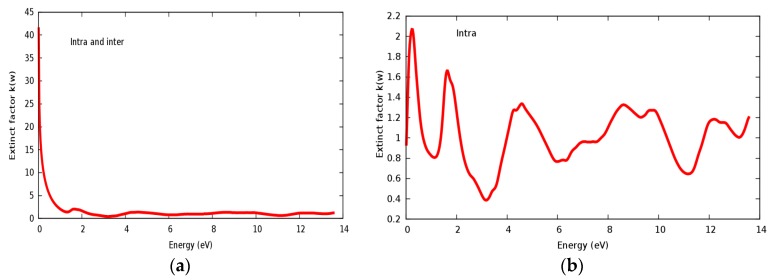
(**a**) Extinct factor *k*(ω) within inter and intra; (**b**) Extinct factor *k*(ω) within intra for SrRhO_3_ compound.

The refractive index *n*(ω) and extinct factor *k*(ω) for the SrRhO_3_ compound are illustrated in Figure 11a,b and Figure 12a,b, respectively. The SrRhO_3_ has a high *n*(0) and *k*(0) with intra and inter band transition, which indicates metallic behavior for the real and imaginary parts of the dielectric. As the incident light energy increases the *n*(ω); and *k*(ω) goes to a lower values. Many peaks are shown in *n*(ω) and *k*(ω) spectra, these peaks originate from intra-band transition. The extinction coefficient depends on the amount of absorption of the photon when it propagates in the material, while the refractive index indicates the phase velocity of the electromagnetic wave.

**Figure 13:**
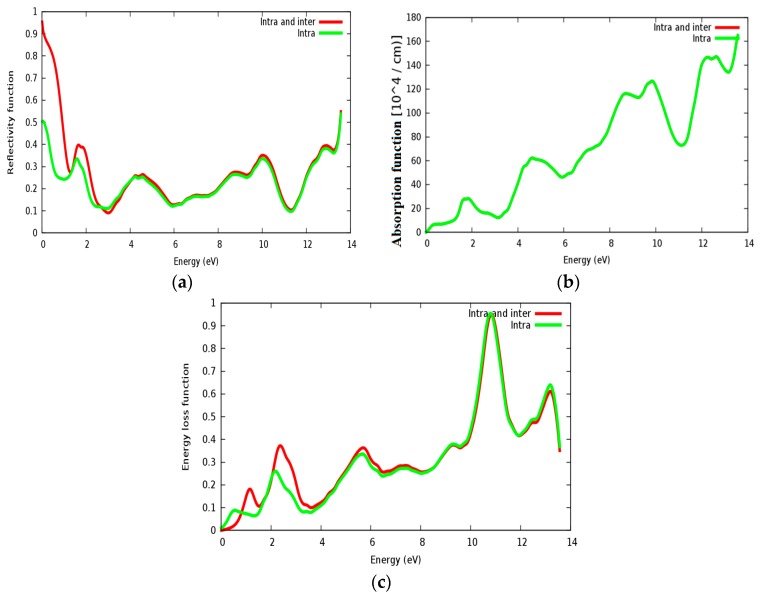
(**a**) Reflectivity function *R*(ω); (**b**) absorption function; and (**c**) energy loss function *L*(ω) for SrRhO_3_ compound.

The reflectivity spectra of SrRhO_3_ as a function of energy are shown in Figure 13a. The static reflectivity of SrRhO_3_ within intra and inter transition is 0.95, while it is 0.5 within intra transition. The reflectivity of SrRhO_3_ in the low energy region goes down as the incident light energy increases, while it increases in the high energy region—the far ultraviolet region (FUV). The absorption coefficient spectra of SrRhO_3_ is plotted in Figure 13b, absorption spectra, as shown, begins at the early beginning and increases as the incident photon energy increases with some peaks along the spectrum. The observed peaks in the spectra related to electron transitions from conduction to valence bands, sharp peaks in the absorption spectrum may be accordance with transitions between valance and conduction band (inter band transitions) that can be considerably far from each other. The SrRhO_3_ compound is a good absorbent compound because it is a metal compound and it is identical with intra + inter and intra band transition. Peaks in the spectrum of the absorption coefficient are proportional to the peaks in the (ε_2_) spectrum. The energy loss function *L*(ω) is describing the energy loss of the fast electrons that propagate inside the material. The energy loss spectrum of SrRhO_3_ is depicted in Figure 13c. Energy loss for SrRhO_3_ is high along the whole spectrum and it is identical with intra + inter and intra band transition in the high energy region. We observe some peaks, the highest peaks are related to the plasma frequency [50]. From these Figures, the plasma frequency of SrRhO_3_ occurs at 10.9 eV and 13.2 eV.

**Figure 14:**
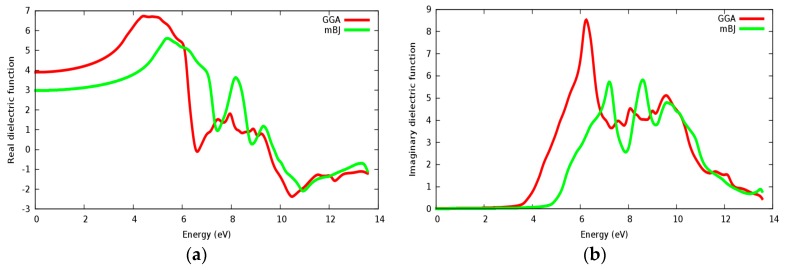
Dielectric function for SrZrO_3_: (**a**) Real part; (**b**) Imaginary part using both PBE-GGA and mBJ-GGA methods.

Figure 14a,b displays the calculated real and imaginary parts of the dielectric function for the SrZrO_3_ compound for a radiation up to 14 eV. It is seen that the calculated linear optical components ε_1_(ω) and ε_2_(ω) spectra for SrZrO_3_ are different from SrRhO_3_. The calculated ε_2_(ω) spectra show that the first critical point (threshold energy) of the dielectric function occurs at about 4.52 eV within the mBJ approach (GGA: 3.6 eV) for SrZrO_3_; the first critical points are comparable with the energy band gap computed from the energy band structure. We can see from Figure 14b, that ε_2_(ω) displaced to the high energy region within the mBJ approach, but the first peak in ε_2_(ω) within the GGA approach has a higher value. The threshold’s energy is followed by some peaks that originate from direct optical transition between the valence band and conduction bands. The main peak in the absorptive spectra is positioned at about 7.2 eV within mBJ (GGA: 6.2 eV). The real part ε_1_(ω) gives us information about the material’s polarizability; the static dielectric constant ε_1_(0) of SrZrO_3_ is 3 within the mBJ approach (GGA: 3.92), as we can see that the mBJ approach has a lower value of ε_1_(0). The value of ε_1_(ω) within mBJ displaced to the higher energy region along the spectrum. The behavior with the high dielectric constant makes SrZrO_3_ a possible useful candidate for manufacturing high capacitors [51]. The negative value in the spectra of ε_1_(ω) for the SrZrO_3_ compound is located only in the ultraviolet spectrum; and shows the metallic behavior of this compound in the mentioned region.

**Figure 15:**
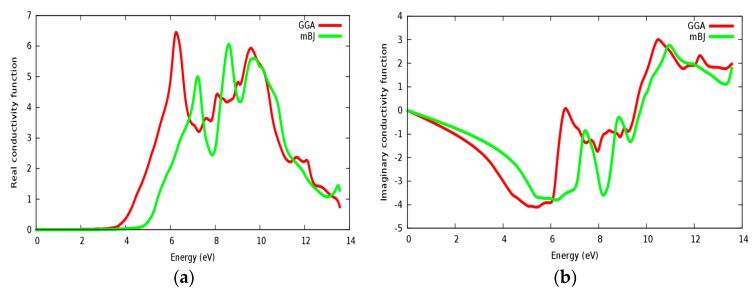
Conductivity function for SrZrO_3_: (**a**) Real part; (**b**) Imaginary part using both PBE-GGA and mBJ-GGA methods.

In Figure 15a,b, the real and imaginary parts of conductivity are illustrated for SrZrO_3_, respectively. It can be seen from Figure 15a, that the real part of conductivity starts to have considerable values at about 4.75 eV within the mBJ approach (GGA: 3.6 eV). From the conductivity and imaginary part of the dielectric function spectrums (Figure 14b), it is shown that both of them start to have a considerable value from approximately the same value. We have also observed some peaks in the conductivity spectra as also observed in the ε_2_(ω) spectra. The conductivity increases as the material becomes more photon (energy) absorbent.

**Figure 16:**
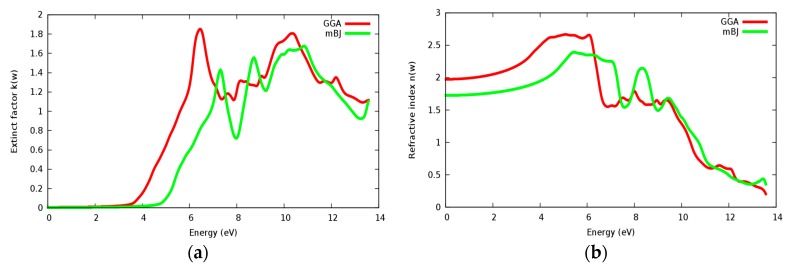
(**a**) Extinct factor *k*(ω); (**b**) Refractive index *n*(ω) for SrZrO_3_ compound using both PBE-GGA and mBJ-GGA methods.

Figure 16a,b displaying the extinction coefficient and refractive index *n*(ω). One can remark that the extinction coefficient of SrZrO_3_ starts to have considerable value at 4.75 eV within mBJ (GGA: 3.6 eV). We can clearly see that *k*(ω), conductivity and ε_2_(ω) start to have considerable values at the same point. Some peaks are presented along the spectrum, these peaks are related to the electrons transitions from valence to conduction bands. It is clearly seen that the extinction coefficient and the imaginary part of the epsilon vary in the same way. The static value of the refractive index *n*(0) in Figure 16b for the SrZrO_3_ compound is 1.75 within the mBJ approach (GGA: 1.9), which is a small value compared to *n*(0) for the SrRhO_3_ compound.

**Figure 17:**
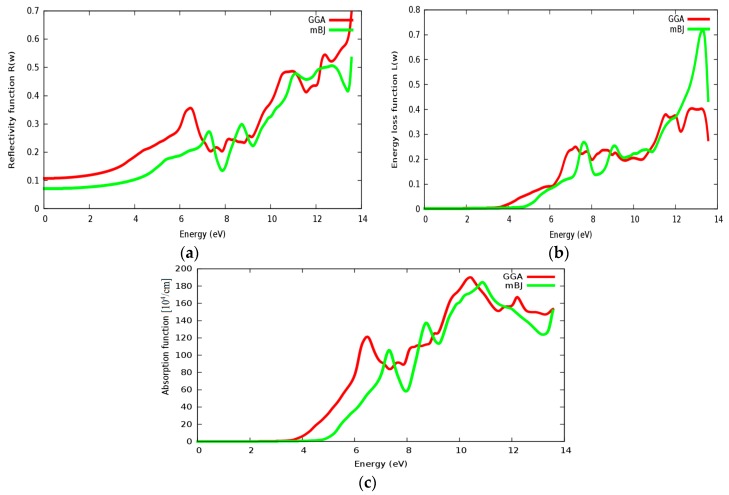
(**a**) Reflectivity function *R*(ω); (**b**) Energy loss function *L*(ω) and (**c**) absorption function for SrZrO_3_ compound using both PBE-GGA and mBJ-GGA methods.

Reflectivity, energy loss and absorption functions are illustrated in Figure 17a–c, respectively. The static reflectivity of SrZrO_3_ is about 0.07 within the mBJ approach (GGA: 0.11). The static reflectivity of SrRhO_3_ is more than nine times greater than the reflectivity of SrZrO_3_. The reflectivity value increases rapidly in the high energy region; far ultraviolet region (FUV); *ab* = *nd* for the SrZrO_3_ compound, indicating that SrZrO_3_ is suitable as a wave reflectance compound in the far ultraviolet region (FUV). Energy loss and absorption functions are seen in Figure 17b,c; both of them quietly behave in the same way. Both absorption and energy loss for SrZrO3 begin at about 4.8 eV within the mBJ approach (GGA: 3.8 eV). The SrZrO_3_ is a good absorbent compound, but not in the low energy region; it is good absorbent in the far ultraviolet region, as it shows metallic behavior in the high energy region.

## 4. Conclusions

In summary, we have studied the structural, electronic, elastic and optical properties of SrTMO_3_ (TM = Rh, Zr) using the FP-LAPW method within the PBE-GGA and mBJ-GGA approaches in the framework of density functional theory. The lattice parameter is found to be in good agreement with experimental and theoretical results. From the band structure, we found that the SrRhO_3_ compound has a metallic nature within both BPE-GGA and mBJ-GGA approaches, while SrZrO_3_ is found to be an insulator within mBJ-GGA, while it is a wide energy band gap semiconductor within PBE-GGA. The elastic constants (*C*_11_, *C*_12_, *C*_44_), bulk modulus, shear modulus and Young’s modulus are also calculated and discussed. By analyzing the *B*/*S* ratio and Poisson’s ratio, we found that SrRhO_3_ has a ductile nature, while SrZrO_3_ has brittle nature. SrZrO_3_ has a more ionic trend compared to the SrRhO_3_. The optical properties such as dielectric constant, absorption coefficient, reflectivity coefficient, refractive index, optical conductivity and energy loss function were investigated in the energy range (0–14) eV. According to the dielectric constant, SrZrO_3_ has a large static dielectric constant which may make it promising as a good dielectric material; while SrRhO_3_ has a negative real dielectric constant which indicates a metallic behavior. It would be interesting to make detailed measurements of the transport and thermodynamic properties of SrRhO_3_ under applied fields looking for metamagnetic behavior and non-Fermi-liquid scalings. In conclusion, our theoretical calculations on SrTMO_3_ (TM = Rh, Zr) will help to use these materials for practical applications.

## Figures and Tables

**Table 1:** Calculated lattice parameter (*a*), bulk modulus (*B*), first pressure derivative (*B*’) of SrTMO_3_ (TM-Rh, Zr).

Compound		*a* (Å)	*B* (GPa)	*B*’
SrRhO_3_	Present work	3.976	162.55	5.97
	Experimental work	3.92 [23]	–	–
	Other theoretical work	3.932 [12] 4.074 [14]	–	–
SrZrO_3_	Present work	4.176	153.25	3.98
	Experimental work	4.109 [24]	150 [25]	–
	Other theoretical work	4.076 [12] 4.177 [14]	–	–

**Table 2:** Calculated energy band gap *E*_g_ (eV) of SrZrO_3_ compound.

Present Work		Other Theoretical Work
PBE-GGA	mBJ-GGA	
3.69 eV	4.53 eV	3.37 eV [26]

**Table 3:** Calculated elastic constants of SrTMO_3_ (TM = Rh, Zr).

Compound		*C*_11_	*C*_12_	*C*_44_	*B*	*S*_H_	*B*/*S*_H_	*Y*	*ν*	*A*
SrZrO_3_	Present work	319.61	72.54	88.05	154.90	102.2	1.52	248.62	0.23	0.71
	Theory [38]	338.60	71.00	77.00	160.00	118.8	1.35	247.64	0.20	0.58
	Theory [14]	299.16	72.57	72.58	124.76	74.81	1.67	241.34	0.25	0.64
SrRhO_3_	Present work	239.53	131.17	100.27	167.29	78.32	2.14	203.25	0. 30	1.85
	Theory [14]	196.25	99.9	46.28	132.01	47.02	2.81	126.11	0.34	0.96
